# The Cape Town International Convention Centre from the inside: The family physicians’ view of the ‛Hospital of Hope’

**DOI:** 10.4102/phcfm.v12i1.2667

**Published:** 2020-10-26

**Authors:** Steve Reid, Tasleem Ras, Klaus von Pressentin

**Affiliations:** 1 Primary Health Care Directorate, Faculty of Health Sciences, University of Cape Town, Cape Town, South Africa; 2 Division of Family Medicine, Faculty of Health Sciences, University of Cape Town, Cape Town, South Africa

**Keywords:** COVID-19, Field Hospital, Cape Town, CTICC, Hospital of Hope

## Abstract

This short report captures the week-by-week reflections of a group of family physicians who joined the clinical and operational management teams tasked with providing the in-patient service of an 862-bed COVID-19 field hospital. The ‘Hospital of Hope’ at the Cape Town International Convention Centre (CTICC) was established as an intermediate care facility specifically to cope with the effects of the COVID-19 pandemic in Cape Town metropole. In an extraordinary feat of engineering, the conference centre floor was transformed within a matter of weeks into wards with piped oxygen at each bed. Whilst the emergency medicine specialists took the lead in designing and commissioning the facility, the medical management and staff were drawn mostly from family physicians. This report is a short reflection on the experience of the first 4 weeks of managing patients in this repurposed space. Our insights evolved during various formal and informal learning conversations as the in-patient service became more organised over time. We hope that these insights, as well as the process of reaching them, will assist other colleagues in serving their communities during this difficult moment in history; moreover, it may reflect a renewed appreciation for team-based interdisciplinary efforts in achieving person-centred care.

## The first week

The first week, launched by the first patient arriving on Monday 8th June 2020, was both exhilarating and challenging, as the results of weeks of frenzied preparation by engineers, funders, planners and managers were finally realised. The infrastructure itself is extraordinarily well planned and assembled, with piped oxygen available at every single one of the 862 beds, all neatly laid out in open ‘wards’ of around 30-beds each. Enormous attention has been given to the flow of patients, staff and supplies to minimise the chances of spread of infection, bearing in mind that hospitals have been shown to be one of the major sites of spread in other countries. There are three tiers of space: ‘outside’ including an area in the underground car park for changing into scrubs, the non-clinical area including the canteen and admin offices where people just use masks and the clinical area, which is entered via a specific room for donning personal protective equipment (PPE).

There were initially six teams (later eight) each with five to six medical officers, with the team leaders drawn from emergency medicine (two), internal medicine (one) and family medicine (six). The first week was surprisingly easy, on reflection by the medical staff, and they felt well prepared as a team, despite a steep learning curve. It helped that there were fewer patients than initially expected in the first week (50 instead of the anticipated 60) as the referral systems bringing patients in were new. The positive attitudes of all the staff stemmed from the fact that they had chosen to be there and came with a strong sense of commitment and wanting to contribute. There was a sense of interdependence and mutual trust, in that ‘we are all in this together’, extending to all categories of staff including admin and cleaners. The medical consultant team expressed their positions at the end of the first week in terms of feeling ‘thankful’, ‘blessed’ and ‘a privilege to be here’.

At the same time, there is also a sense of anxiety and pressure to get things done and systems sorted out, with the expected massive escalation in the numbers of patients looming. Although most of these speed bumps were dealt with rapidly, one of the clinical managers said he felt ‘anxious most of the time’.

## The second week

The second week (starting 15th June 2020) was characterised by getting systems in place in anticipation of an inevitable surge of patients. With very new staff, including the addition of 18 Cuban doctors, there was a need to rearrange ways of working to be more efficient.

There were still fewer numbers of patients than expected, admitting around 20 to 30 per day, but quite a good discharge rate too. This was a week of testing the systems and a routine started to form around the ward work, with morning team huddles, lunchtime consultant meetings, visiting consultants from the teaching hospitals and the operational management team holding their weekly learning session.

The first morbidity and mortality (M&M) meeting was held, with an excellent presentation reflecting on a particularly challenging patient who had to be moved to ICU at Groote Schuur and who unfortunately died there. This experience tested the new teams and the referral systems and raised questions about how COVID-19 causes pathology such as thrombosis, with the following lessons being learnt:

1. Being aware of ‘COVID blinkers’, assuming that every problem is because of COVID-19.
2. Expect sudden rapid deteriorations.
3. Trust your intuition – submitting immediate decisions (‘fast’ thinking) to later review (‘slow’ thinking).^1^

Later in the week, the operational management team articulated the main aim and the objectives of the hospital as follows:

To offer hope by delivering high quality and efficient in-patient care in response to the needs in the metropole, whilst ensuring the safety and positive growth of staff.

This balance of the care of patients and the safety of staff is a crucial and continuous tension that has to be managed.

The primary objectives to achieve the aim are as follows:

1. To deliver evidence-based, safe, comprehensive patient-centred care.
2. To ensure the safety and positive professional growth of staff and teams.
3. To manage the facility efficiently.
4. To collaborate effectively with other health facilities in the metropole.
5. To promote system learning – not only internally but also in the whole metropole.

## The third week

Suddenly things got serious in the week (22nd June 2020), with a jump in the number of patients admitted from 40 to 50 each day and discharged patients were slightly less, such that by the end of the week we had 220 patients in the hospital. Accordingly, staff and systems became stressed, and the added pressure to optimise system efficiency, corresponding with the third objective ‘to manage the facility efficiently’. With a new and complex system, this proved more difficult than it seemed. Issues that needed to be clarified included the criteria for admission (mostly sorted through the Vula app that facilitates referrals), the availability of scrubs and PPE (ongoing), staffing and trouble-shooting over weekends, communication between patients and their families, the discharge process. With a number of expected deaths, there was also a need for a standard operating procedure (SOP) for dealing with a death and the corpse.

On the positive side, there was encouraging feedback from discharged patients and relatives, that they had felt cared for, despite the frustrations of communication from the clinical area. The acute platform’s feedback about the impact that we were having on de-stressing their bed occupancy was also a source of encouragement. On a clinical level, the introduction of prednisone or dexamethasone based on research published in the United Kingdom the previous weekend, seemed to make a difference to some oxygen-dependent patients within a day or two.

We started looking at improvement processes and introduced the idea of incremental improvement starting with a ‘good enough’ SOP, so that everyone had something to work with initially and could add to it or change it over time. The discharge process, for example, came under scrutiny as it was haphazard and disorganised. But just by bringing a team of people together with representatives from each section involved including nursing, pharmacy, transport and the doctors, it proved relatively easy within a few days to streamline the process to get all patients identified for discharge to the transport on time.

## Week four (29th June 2020)

This week the numbers of patients escalated to over 800 admissions since opening. We had 322 patients in the wards at the end of week four, so the discharges were also frequent, indicating that most patients got better. There were 24 deaths by the end of the month. At the M&M meeting, we celebrated the teamwork that came together around a very sick patient who needed to be intubated before emergency referral to the tertiary hospital. This meeting, with the input of an ethicist from the university, generated a discussion on the ethical dimensions of care, which clarified some of the difficult decisions around escalating or withholding care and focussed on the care pathway to ICU for these critically ill patients.

This is a very biomedical space deciding on who should get into ICU is not something we normally do. As one of the family physicians said, we are usually talking to recalcitrant diabetics about adhering to their treatment, rather than deciding on who gets the lucky ticket to tertiary care. And this is where the challenge lies for us particularly as family physicians – how to mitigate the effects of this industrial-level response to a single disease that has been forced upon us by sheer weight of numbers and turn it into an opportunity for human connection and meaning. Despite the barriers to PPE and the factory-like environment, the doctors are still able to connect with patients on a human level at the bedside, ask about their homes, explain the disease, help them to understand the oxygen saturation percentage or connect them with their family members by video calls. With one particularly ill patient on oxygen, it was observed that his oxygen saturation levels rose significantly after a video call to his family. How would anaesthetists explain that?

## Reflections

**FIGURE 1:**
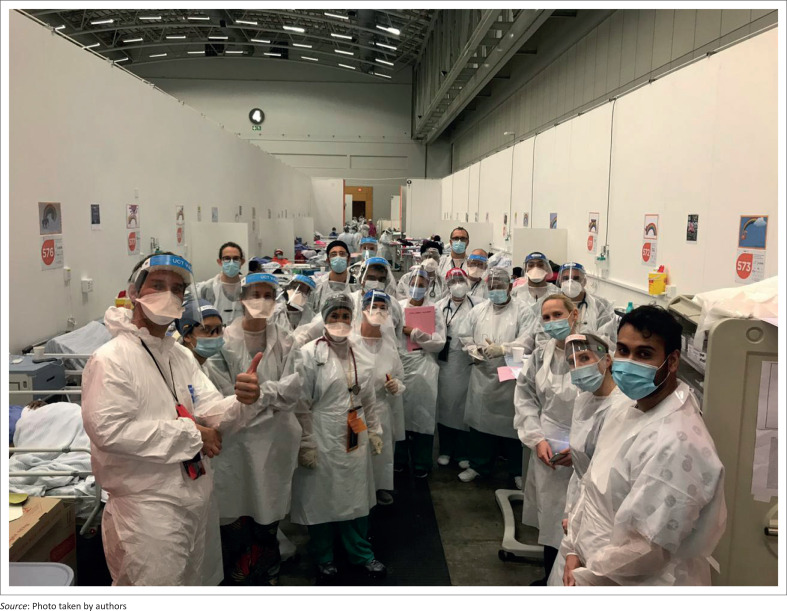
Medical officers and team leaders on ward round with a visiting consultant at the Hospital of Hope.

Reflecting on the first month of operation of the Hospital of Hope as family physicians, we realise the significant value of close teamwork and frequent communication, with clear leadership and planning, but above all the willingness to adapt and learn (see Figure 1). With an entirely new hospital and staff complement, it was possible to create an excellent team from scratch based on the selfless commitment of the staff who signed up despite the risks. A month later we are now developing indicators to assess whether we are achieving our objectives. Although hope, which was articulated as the overall aim of the hospital, is impossible to measure as such, there is no doubt in our minds (and hearts) that this is what keeps us coming in to work every day (see Box 1).

BOX 1 Ten do’s and don’ts for setting up a field hospital in South Africa.

**Based on our experiences at the Cape Town ICC ‘Hospital of Hope’ we offer the following advice to others who areinvolved in setting up similar field hospitals in South Africa**:

1. Do as much planning as possible before accepting the first patient with respect to engineering, supplies and systems.
2. Assemble a management team that draws on experience from other hospitals and is prepared to regularly walk around inPPE on the floor.
3. Make sure that oxygen is available at each bedside – this is the most precious commodity. Without oxygen, it is just anisolation facility.
4. Keep protocols as simple as possible (e.g. diabetic regimes – use protophane once daily for BS control <12mmol/L). Don’ttry and make it perfect.
5. Start with ‘good enough’ SOPs for common processes, for example, discharge, deaths, escalations that can be improvedover time. Don’t wait for someone higher-up to sign them off.
6. Put multidisciplinary teams in place from the start – a set number of nurses, doctors, physio’s for a fixed number ofpatients in a ward. Don’t develop the doctors teams separately from the nursing teams.
7. Maintain continuity of care as far as possible – this hugely increases efficiency. Don’t move patients or nurses around allthe time.
8. Make critical decisions as a team: don’t make decisions alone (e.g. high flow nasal oxygen or referral to ICU).
9. Have an exit plan for each patient at admission (e.g. for ICU if necessary or palliation)
10. Liaise daily with hospitals referring patients in, to assess their need and let them know of your capacity.
